# Rheological properties of blood in multiple myeloma patients

**DOI:** 10.1038/s41598-024-54947-4

**Published:** 2024-02-21

**Authors:** Bartłomiej Ptaszek, Szymon Podsiadło, Zuzanna Jandziś, Aneta Teległów, Anna Piotrowska, Artur Jurczyszyn, Olga Czerwińska-Ledwig

**Affiliations:** 1 Institute of Applied Sciences, University of Physical Education in Krakow, 31-571, Kraków, Poland; 2 Institute of Clinical Rehabilitation, University of Physical Education in Krakow, 31-571, Kraków, Poland; 3 Faculty of Motor Rehabilitation, University of Physical Education in Krakow, 31-571, Kraków, Poland; 4 Institute of Basic Sciences, University of Physical Education in Krakow, 31-571, Kraków, Poland; 5 Department of Hematology, Jagiellonian University Medical College, 31-501, Kraków, Poland

**Keywords:** Blood rheology, Red blood cell, Red blood cell deformability, Red blood cell aggregation, Multiple myeloma, Biochemistry, Biomarkers, Oncology, Pathogenesis

## Abstract

Multiple myeloma (MM) is considered to be one of the hematological malignancies formed by excessive and abnormal proliferation of plasmocytes. Among other parameters, several blood tests are used to diagnose multiple myeloma. The hemorheological profile in multiple myeloma is not widely studied. Hemorheology includes the study of measuring the deformability and aggregation of erythrocytes, blood viscosity, and sedimentation rate. The degree of deformability of blood cells is necessary to maintain proper vital functions. Proper deformability of red blood cells ensures proper blood circulation, tissue oxidation and carbon dioxide uptake. The aim of the study was to compare morphology and blood rheology parameters in patients with MM and healthy individuals. The study included 33 patients with MM, and 33 healthy subjects of the same age. The hematological blood parameters were evaluated using ABX MICROS 60 hematology analyzer. The LORCA Analyzer to study erythrocyte aggregation and deformability. Patients with MM had lower red blood cells count (RBC) (9.11%) (p < 0.001) and half time of total aggregation (T1/2) (94.29%) (p < 0.001) values and higher mean corpuscular volume (MCV) (5.50%) (p < 0.001), aggregation index (AI) (68.60%) (p < 0.001), total extent of aggregation (AMP) (87.92%) (p < 0.001) values than the healthy control group. Aggregation in patients with MM is different compared to healthy individuals. It was observed that the percentage of cell aggregation is almost 50% higher than in the control group. The study of morphology, aggregation and deformability of erythrocytes in patients with suspected MM may be helpful in making clinical decisions.

## Introduction

Multiple myeloma (MM) is a malignant neoplasm of the hematopoietic system. It is formed by uncontrolled proliferation of plasmocytes in the bone marrow. In the diagnosis of patients, blood count, blood smear and biochemical tests such as electrophoresis of plasma proteins or monoclonal protein detection in serum or urine should be performed^1^. The main symptoms of multiple myeloma include bone pain, located most often in the pelvic and lumbar spine area^2^, pathological fractures, which is associated with bone marrow failure, and increased perceived fatigue. Excessive blood viscosity, anemia, kidney disease and hypercalcemia can also suggest the diagnosis of MM. Diagnosis is based on CRAB and SLiM criteria^3^. Globally in 2018, multiple myeloma accounted for 0.9% of all cancer diagnoses^4^. Risk factors include black race, age over 50, male gender, overweight and obesity, and exposure to carcinogens^5^.

Applied MM treatment depends on the age of the patients and overall condition. The main treatment procedures are autologous cell transplantation, induction therapy and consolidation therapy. Immunotherapy methods are a new methods of treatment, the most promising of which is CAR-T cell therapy^5^. Patients younger than 70 years of age and in overall good condition are eligible for myeloablative therapy (high dose therapy, HDT) together with autologous hematopoietic cell transplantation (auto-HSCT)^6^. Patients ineligible for HDT are treated based on low-dose melphalan-based protocols with the addition of newer drugs. The main groups of drugs used in treatment are alkylating agents, corticosteroids, immunomodulatory drugs and proteasome inhibitors^7^. The use of physiotherapy in patients with myeloma results in a reduction in perceived fatigue^8^ as well as can affect patients’ functional ability^9^. In 2013, a study was conducted that found improvements in quality of life and strength after the intervention. The therapy process included stretching, aerobic and resistance exercises and lasted for a total of 6 months^10^. Research by Czerwińska et al. showed that for patients with MM, Nordic walking training is a safe form of physical activity, and could positively affect serum vitamin D concentration^9^, as well as disease-related blood parameters^11^.

The most commonly observed abnormality in blood cells morphology examination found in MM patients is anemia. Also, the erythrocyte sedimentation rate is usually significantly increased. Creatinine and calcium concentrations are also elevated^1^.

Rheology is a science that describes the change in shape of materials and the flow of substances under the action of forces. Hemorheology is a sub-discipline that studies the behavior of blood cells and blood vessel walls due to the pressure of flowing blood. In hemorheology studies, multiple myeloma patients show an increase in plasma viscosity and a decrease in hematocrit. A decrease in erythrocyte deformability has also been noted^12^. It is necessary to confirm the existing research results in this topic. The aim of this study was to investigate and evaluate blood morphological and rheological indices in patients with MM and healthy subjects.

## Material and methods

### Ethical approval

The presented study was conducted in accordance with the Helsinki Declaration of the World Medical Society; and the study was approved by the Bioethics Committee at the Regional Medical Chamber in Krakow (166/KBL/OIL/2018). This study was also registered as a clinical trial in ANZCTR (no: ACTRN12622000268741; date registered: 14/02/2022).

### Participant characteristics

The study involved 33 patients with MM (17 women, 16 men) in remission stage (comorbidities: polyneuropathy n = 14, bone pain n = 22, myeloma bone disease (MBD) n = 30). The control group consisted of 33 healthy people (18 women, 15 men) without hematological diseases and other chronic diseases of the same age as the participants in the study group. Patients from the control group did not report any comorbidities. Study group characteristics is shown in Table 1. During the project, the participants were under the medical care of a hematologist and physiotherapist. During the project, the patients did not change their eating style and did not participate in other forms of physical activity. Volunteers gave written consent to participate in the study.

**Table 1:** Study group characteristics.

Characteristics	Multiple myeloma patients (MM, n = 33)	Healthy control (CONT, n = 33)	*p*
Age [years]	63 ± 6	65 ± 5	0.151
Body height [cm]	166 ± 9	162 ± 4	0.119
Body mass [kg]	79.8 ± 12.3	74.04 ± 8.7	0.092
Body mass index [kg/m^2^]	28.8 ± 3.6	27.4 ± 2.6	0.129
Time from diagnosis [months]	35.7 ± 15.8	–	–

Data is shown as average ± standard deviation (SD). Statistically significant results were considered for *p* < 0.05. No statistically significant differences were found between groups.

MM: multiple myeloma; BMI: body mass index.

### Analysis of blood parameters

For the analysis of studied blood parameters, venous blood was collected once from each group of patients. Blood was collected from the subjects on an empty stomach in the morning (08:00–09:00 A.M.) from the antecubital, cephalic, or median vein into test tubes with EDTA K2 (4 ml) (with a tourniquet) at normal ambient temperature after a 10 min resting period and in a seated position. The blood was collected by a qualified laboratory diagnostician, in accordance with the applicable standards^13^.

Assessment of hematological parameters of the blood was done using the ABX MICROS 60 hematology analyzer (USA). The LORCA analyzer (Laser–Optical Rotational Cell Analyzer, RR Mechatronics, the Netherlands) was used to study the aggregation and deformability of erythrocytes, and the results were given as the elongation and aggregation index (EI and AI). Tests in the aforementioned device were performed within 30 min of blood collection, at 37 °C, according to a standard protocol^13–16^.

The blood used for determining the elongation index was taken from test tubes in the amount of 25 μL to 5 mL of 0.14 mM PVP (Polyvinylpyrrolidone), dissolved in a buffered saline solution (PBS). The test sample was placed in the measuring chamber between two concentric cylinders which were set in rotation. The laser light, passing through the thin layer of red blood cells suspended in the PBS solution, was deflected, giving a diffraction image on the projection screen. The EI results are given in the range from 0.30 to 60.30 shear stress measured in Pascals (SS). EI is a measure of the amount of deformation of red blood cells as they move through the measurement chamber. The blood sample, prior to the actual aggregation test, was oxidized by incubation and mixed with carbogen for 15 min. Then, 1.5 mL of blood was introduced into the measuring chamber of the LORCA analyzer. The cylinder was rotated within 120 s with a shear rate of > 400 s^−1^. After 10 s, the centrifugation stopped abruptly, and the aggregation of red blood cells began. The result of the computer analysis represents the time dependence of the scattered light intensity (for a specific shear rate), i.e., selectogram. Parameters Determining the Kinetics of Erythrocyte Aggregation Were Investigated: AI (%) (Aggregation Index), AMP (au) (Total Extend of Aggregation), T 1/2 (s) (Half Time Kinetics of Aggregation)^14–16^.

### Statistical analysis

Descriptive statistics were determined for every variable: mean (x) as well as standard deviation (SD). The normality of distributions was verified with the Shapiro–Wilk test. Data analysis was performed using parametric tests – the Student’s t-test for independent samples performing comparisons between the groups. The applied tests verified two-sided hypotheses. The analyses were performed with the use of the Statistica 13 package (Tibco Software Inc., USA).

### Informed consent statement

Informed consent was obtained from all subjects involved in the study.

## Results

**Figure 1:**
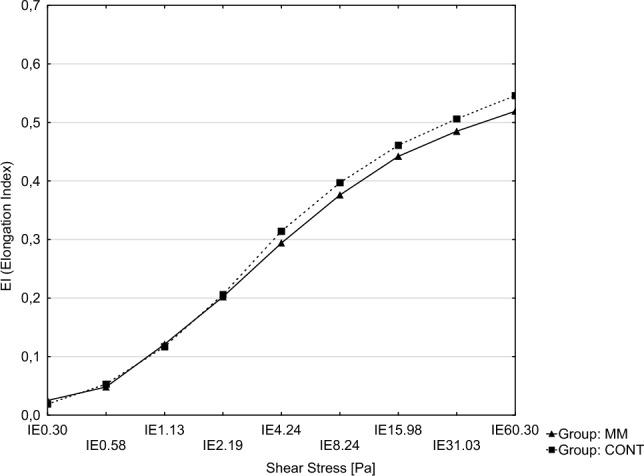
Elongation Index in the experimental group (MM) and in the control group (CONT).

Observed blood cells count values were within the reference ranges in both groups. In patients with multiple myeloma, statistically significantly lower values of RBC and higher values of MCV were observed than in healthy controls. In studied hemorheological parameters in MM patients we have observed statistically significantly lower values T1/2 and higher values of AI and AMP. Results are shown in Table 2 and Fig. 1.

**Table 2:** Morphological and rheological indices of blood in the experimental group (MM) and in the control group (CONT).

Parameter	MM (n = 33)	CONT (n = 33)	*p*
WBC (10^9^/L)	5.31 ± 1.31	5.71 ± 2.03	0.350
RBC (10^12^/L)	4.19 ± 0.54	4.61 ± 0.47	< 0.001*
HGB (g/dL)	13.14 ± 1.52	13.37 ± 1.45	0.541
HCT (%)	38.23 ± 5.73	40.20 ± 4.31	0.127
PLT (10^9^/L)	226.42 ± 65.40	246.74 ± 58.44	0.196
MCV (fl)	92.06 ± 4.35	87.26 ± 4.99	< 0.001*
MCH (pg)	31.49 ± 1.97	30.97 ± 8.16	0.722
MCHC (g/dL)	33.97 ± 2.68	33.23 ± 0.60	0.142
EI 0.30	0.035 ± 0.058	0.022 ± 0.017	0.218
EI 0.58	0.070 ± 0.091	0.059 ± 0.023	0.507
EI 1.13	0.121 ± 0.028	0.125 ± 0.028	0.558
EI 2.19	0.209 ± 0.031	0.214 ± 0.038	0.626
EI 4.24	0.302 ± 0.033	0.304 ± 0.055	0.887
EI 8.24	0.379 ± 0.038	0.377 ± 0.074	0.864
EI 15.98	0.442 ± 0.043	0.443 ± 0.074	0.928
EI 31.03	0.484 ± 0.048	0.487 ± 0.078	0.849
EI 60.30	0.517 ± 0.049	0.520 ± 0.078	0.834
AI (%)	65.08 ± 6.95	38.60 ± 25.36	< 0.001*
AMP (au)	22.87 ± 3.16	12.17 ± 13.11	< 0.001*
T1/2 (s)	1.97 ± 0.83	34.52 ± 30.17	< 0.001*

Data is shown as average ± standard deviation (SD). Statistically significant results were considered for *p* < 0.05 [*].

WBC: white blood cells count; RBC: red blood cells count; HGB: hemoglobin concentration; HCT: hematocrit, PLT: platelet count; MCV: mean corpuscular volume; MCH: mean corpuscular hemoglobin; MCHC: mean corpuscular hemoglobin concentration; EI: elongation index for shear stress value between 0.30 and 60.30; AI: aggregation index; AMP: total extent of aggregation; T1/2: half time of total aggregation.

## Discussion

Multiple myeloma is a cancer of plasma cells that accumulate in the bone marrow, leading to bone deterioration and bone marrow insufficiency. Bones, kidneys and bone marrow are mainly impacted by multiple myeloma^17^. The anemia that often occurs during myeloma is related to the suppression of the erythropoiesis pathway related with uncontrolled proliferation of plasmocytes, as is the leukopenia that occurs. The production of monoclonal protein by plasmocytes in MM patients causes a lot of adverse changes occurring in various body organs. It has already been indicated that patients may have disorders related to the rheological properties of the blood caused by the presence of the monoclonal protein, such as hyperviscosity syndrome causing microcirculatory disorders^18^. It also appears that the properties of erythrocytes may be altered in these patients. In this study we have involved 33 MM patients in remission stage, after anti-myeloma therapy, whose disease-related blood parameters levels were stable. Therefore, the aim of the study was to investigate and evaluate the morphological and rheological indicators of blood in patients with multiple myeloma and healthy individuals.

Hemorheology includes the study of measuring blood viscosity, sedimentation rate (RBS), deformability and aggregation of erythrocytes. The degree of deformability of blood cells is an essential for maintaining normal vital functions. Proper deformability of red blood cells ensures proper blood circulation, tissue oxidation and carbon dioxide uptake^19^.

In this study, reduced RBC and MCV levels have been observed in the morphology of patients with multiple myeloma compared to control group. A study of Indrarsi and Sukorini (2020) also found visibly reduced erythrocyte levels, while no change in MCV was observed^20^. In a study conducted by Al Saleh et al. (2020), the MCV level was approximately the same as in our study. The researchers proved that the hematopoietic score can imply survival in newly diagnosed multiple myeloma patients^21^.

As mentioned before, the deformability of red blood cells has a major impact on the blood's ability to circulate^22^. The characteristics of red blood cells such as deformability and preserved aggregation allow proper oxygen saturation of tissues. Reduced deformability of blood cells contributes to reduced blood flow and adequate oxygen concentration in tissues^23^.

Changes rheological properties of blood, including plasma viscosity and erythrocyte deformability, are well known in plasmocytic dyscrasias, including MM and the conditions preceding it^24^. Caimi et al. studied hemorheological properties of blood in 21 patients with monoclonal gammapathy of undetermined significance (MGUS) which is a noncancerous condition preceding myeloma. In this study significantly lower EI values than in control group were found in MGUS patients for various shear forces^25^. In contrast to our study, in which no such differences were found. This may be related to the stage of MM remission the patients were in.

Lemonne et al. have shown in their paper a case of a patient with sickle cell anemia and smoldering myeloma which is a pre-myeloma condition. In this patient a higher AI% than in our study (71.38%). Authors compare values with healthy subjects in which AI is also higher than in our control group (60.7%). Interestingly, in our study EI for shear force at 30 Pa in both groups was similar, while in a cited study—it was significantly lower than in smoldering MM patient. Interpretation of the results in one patient with smoldering myeloma is inconclusive and would require further studies on a wider patient population^26^.

In MM patients, Caimi et al. observed significantly reduced erythrocyte deformability in multiple myeloma 24 patients^12^. In another study of Caimi et al. compared the results of EI in blood samples obtained from 29 MM patients and found out that its values were significantly lower compared with healthy subjects, especially in lower values of shear stress and were not depending on stage of the disease. However, as the study included only 5 patients in remission stage, it is difficult to compare its results with those we received^27^.

Physiological aggregation of red blood cells (rouleaux formation) is a normal phenomenon as long as it occurs in both directions simultaneously and the formation process can be reversed^28^. Increased aggregation and impaired disintegration of the resulting erythrocyte junctions is noticed in disease cases^29^. The problem of RBC increased aggregation in MM patients is long known^30^. It is very often linked with high immunoglobulin concentrations^31^. In our study, we indicated higher erythrocyte aggregation in patients compared to healthy controls. A study by Pribush et al. in which 40 blood samples from MM patients were used indicated that increased erythrocyte aggregation is most likely related to surface-active plasma molecules present in blood^32^. A study by Baskurt and Meiselman noted that increased aggregation of red blood cells is seen in acute circulatory disorders in various organs^28^. Therefore, it is highly significant to monitor blood hemorheology, particularly in multiple myeloma patients, to prevent additional complications. Due to some limitations, this study should be treated as preliminary, mainly due to the small group of patients studied.

## Conclusions

In conclusion, an increase in blood cell aggregation contributes to an increase in plasma viscosity, thereby impairing microcirculation. Factors modulating blood viscosity include plasma viscosity, hematocrit, RBC aggregation and RBC deformability. We have established that performing morphology and erythrocyte aggregation and deformability in patients with suspected MM, supports clinical decision-making. Changes in any of these parameters may contribute to modification of tissue perfusion and resistance to vascular blood flow, hence, as outlined in the article, it is important to study factors such as blood viscosity in rheological clinical disorders.

## Data Availability

All data generated or analysed during this study are included in this published article.
